# Computational studies of the role of serotonin in the basal ganglia

**DOI:** 10.3389/fnint.2013.00041

**Published:** 2013-05-24

**Authors:** Michael C. Reed, H. Frederik Nijhout, Janet Best

**Affiliations:** ^1^Department of Mathematics, Duke UniversityDurham, NC, USA; ^2^Department of Biology, Duke UniversityDurham, NC, USA; ^3^Department of Mathematics, The Ohio State UniversityColumbus, OH, USA

**Keywords:** serotonin, basal ganglia, direct pathway, mathematical model

## Abstract

It has been well established that serotonin (5-HT) plays an important role in the striatum. For example, during levodopa therapy for Parkinson's disease (PD), the serotonergic projections from the dorsal raphe nucleus (DRN) release dopamine as a false transmitter, and there are strong indications that this pulsatile release is connected to dyskinesias that reduce the effectiveness of the therapy. Here we present hypotheses about the functional role of 5-HT in the normal striatum and present computational studies showing the feasibility of these hypotheses. Dopaminergic projections to the striatum inhibit the medium spiny neurons (MSN) in the striatopalladal (indirect) pathway and excite MSNs in the striatonigral (direct) pathway. It has long been hypothesized that the effect of dopamine (DA) depletion caused by the loss of SNc cells in PD is to change the “balance” between the pathways to favor the indirect pathway. Originally, “balance” was understood to mean equal firing rates, but now it is understood that the level of DA affects the patterns of firing in the two pathways too. There are dense 5-HT projections to the striatum from the dorsal raphe nucleus and it is known that increased 5-HT in the striatum facilitates DA release from DA terminals. The direct pathway excites various cortical nuclei and some of these nuclei send inhibitory projections to the DRN. Our hypothesis is that this feedback circuit from the striatum to the cortex to the DRN to the striatum serves to stabilize the balance between the direct and indirect pathways, and this is confirmed by our model calculations. Our calculations also show that this circuit contributes to the stability of the dopamine concentration in the striatum as SNc cells die during Parkinson's disease progression (until late phase). There may be situations in which there are physiological reasons to “unbalance” the direct and indirect pathways, and we show that projections to the DRN from the cortex or other brain regions could accomplish this task.

## Introduction

The serotonergic system projects widely in the brain with targets including sensory, motor, and limbic systems (Feldman et al., 1997; Hornung, 2003), and it participates in many neural functions including cognition, mood, regulation of feeding, and sleep-wake behavior (Feldman et al., 1997; Monti, 2011). The dorsal raphe nucleus (DRN) contains a significant proportion of the brain's serotonergic neurons, and these axons are distinguished from other serotonergic projections by their small varicosities and their lack of true synapses, so that they contribute to volume transmission (Hornung, 2003). The result may be complex modulation of neural activity rather than directly stimulating a specific response, which may account for the fact that the effects of these projections have been long debated; see for example Monti (2010).

The DRN makes dense serotonergic projections to the basal ganglia (BG), including the substantia nigra pars compacta (SNc) and the striatum (Vertes, 1991). While the role of this serotonergic innervation is unclear in healthy individuals, in the past 10 years, it has become appreciated that these serotonergic projections to the striatum play a special role in patients with Parkinson's disease (PD) being treated with levodopa (L-DOPA). In serotonergic neurons, 5-Hydroxy-L-tryptophan is converted to serotonin (5-HT) by amino acid decarboxylase, the same enzyme that converts L-DOPA to dopamine (DA) in dopaminergic cells. Thus, when L-DOPA is systemically administered, as is the common first treatment for PD, serotonergic cells are also able to convert the L-DOPA into DA, store it with 5-HT in the neuronal vesicles, and then release a mixture of 5-HT and DA in response to action potentials (Carta et al., 2007). In the striatum, the implication is that cells are releasing DA without the restraint and homeostatic mechanisms present on DA cells, allowing large fluctuations in the level of extracellular DA in the striatum. When a minority of DA cells have been lost (to PD), the remaining cells may be able to help regulate these fluctuations. But as more DA cells die, these fluctuations are less restrained and dyskinesias occur (de la Fuente-Fernandez et al., 2001; Lindgren et al., 2010). These effects were investigated using a mathematical model in Reed et al. (2012).

In this paper, we present a hypothesis about the role of 5-HT in the healthy basal ganglia, focusing on the effects of 5-HT on the so-called direct and indirect pathways. In the next two sections we describe the direct and indirect pathways and our hypothesis. In the following section, we will present a computational model that we use to test the plausibility of our hypothesis. We will then describe the results of computational experiments on the model.

## Direct and indirect pathways and balance

The BG are a group of subcortical nuclei including the striatum, subthalamic nucleus, internal and external globus pallidus, and substantia nigra. Cortical-BG-thalamic circuits are critically involved in many functions including sensorimotor, emotion, and cognition (Haber and Calzavara, 2009; Lincoln et al., 2012). Multiple paths and subcircuits within BG have been identified. In some cases the different circuits perform different functions; for instance the striatum, the input nucleus of the BG, has anatomic and functional subdivisions including sensorimotor and associative. In other cases, pathways may compete, as has been postulated for action selection.

Two of the most studied pathways through the BG are the direct and indirect pathways, segregated pathways through medium spiny neurons (MSNs) in the striatum. The names reflect the fact that the direct pathway proceeds from the striatum directly to either the internal portion of the globus pallidus (GPi) or the Substantia Nigra pars reticulata (SNr), the two output nuclei of the BG. The indirect pathway, on the other hand, also involves a subcircuit that includes the external portion of the globus pallidus (GPe) and the subthalamic nucleus (STN) before reaching the output nuclei. The two pathways have opposing effects on the thalamus: the indirect pathway has an inhibitory effect, while the direct pathway has an excitatory effect (Smith et al., 1998; Gerfen and Surmeier, 2011).

Albin and DeLong (Albin et al., 1989; DeLong, 1990) proposed that the balance of these opposing pathways is important for healthy function. Dopaminergic cells in the SNc project to the striatum and inhibit MSNs in the indirect pathway and excite MSNs in the direct pathway. Albin and DeLong noted that, during PD, the loss of dopaminergic cells in the SNc has the effect of shifting the balance in favor of the indirect pathway, and they reasoned that the increased inhibitory output from BG to thalamus might account for some of the motor symptoms of PD, such as bradykinesia and difficulty in initiating movement. This view later lost favor in the face of new experimental observations that appeared to contradict the Albin–DeLong theory. The fact that pallidotomy—lesioning the GPi—alleviates some PD motor symptoms fit well with the theory, but it emerged that high frequency stimulation of GPi was equally effective therapeutically. The solution to this conundrum seemed to be that the *pattern* of neuronal firing in the BG was as important for symptoms as the *rate* of firing; it was then often assumed that the Albin–DeLong theory was dead. In particular, it has been established experimentally that firing patterns in the GPi become bursty as PD progresses. Noting that this firing pattern is effectively a stronger signal than the irregular firing observed in the healthy GPi, however, allows the possibility that the Albin-DeLong theory retains merit but the notion of “balance” needs to be interpreted more generally. With this more general notion of balance, it is again widely hypothesized that many of the motor symptoms of PD are due to an imbalance between the direct and indirect pathways (Kravitz et al., 2010; Gerfen and Surmeier, 2011; Zold et al., 2012).

The BG play a critical role in action selection, and it has been proposed that changes in DA levels are important in this process. One key difference between MSNs in the direct and indirect pathways lies in their responses to extracellular DA: MSNs in the direct pathway express D1 receptors and are stimulated by DA while MSNs in the indirect pathway express D2 receptors and are inhibited by DA. It is known that D1 receptors mediate the effect of DA on the dyskinesias mentioned above (Darmopil et al., 2009; Mela et al., 2012). MSNs in both pathways receive feedforward inhibition from cortical pyramidal neurons that project to striatal inhibitory interneurons; this inhibition, together with collateral inhibition from other MSNs, may suppress MSN activity in circuits corresponding to undesired actions. In the circuit of the desired action, selection could depend upon the level of DA. While both the direct and indirect pathways receive the feed forward inhibition, it has been found that these inhibitory projections preferentially connect with the direct pathway and that there is an inhibitory feedback loop from the GPe in the indirect pathway (Bevan et al., 1998; Gerfen and Surmeier, 2011). Since the indirect pathway MSNs express D2 receptors, this feedback loop is expected to be inhibited by basal levels of DA. However, a transient decrease in DA could facilitate the feedback by disinhibiting the inhibitory projection to the GPe. Meanwhile, cortical excitation in the direct pathway helps counter the feed forward inhibition there. In this description of action selection, the presence of DA helps shift the balance in favor of the direct pathway. We mention these details of feedforward and feedback circuits in action selection to show how important the balance between direct and indirect pathways is in considering action selection, but these detailed feedforward and feedback circuits are not in our model.

Computational models of the BG abound, including biophysical models (Terman et al., 2002; Rubchinsky et al., 2003). Many studies focus on functions believed to be performed by the BG (Doya, 1999) such as reinforcement learning (Bar-Gad et al., 2011) or action selection (Gurney et al., 2001; Humphries et al., 2006; Houk et al., 2007; Girard et al., 2008). These models often involve competition between different loops through the BG. Some models explicitly consider the balance between pathways, with a loss of balance hypothesized to occur when DA is depleted (Leblois et al., 2006). Contreras-Vidal and Stelmach (1995) also consider the role of other neuropeptides (dynorphin, Substance P, enkephalin) in the imbalance of pathways that accompanies nigral degeneration. However, these studies do not consider the BG to be embedded in a larger regulatory circuit. Our model uses a simple global circuit as a platform for investigating the role of serotonin in the striatum.

## A hypothesis about the role of 5-HT in the striatum

What is the role of 5-HT in the striatum? Both the discussion of PD and the discussion of action selection above suggest that the balance between the direct and indirect pathways is important for the function of the BG in healthy individuals. We will explain how 5-HT in the striatum may help to maintain that balance.

**Figure 1:**
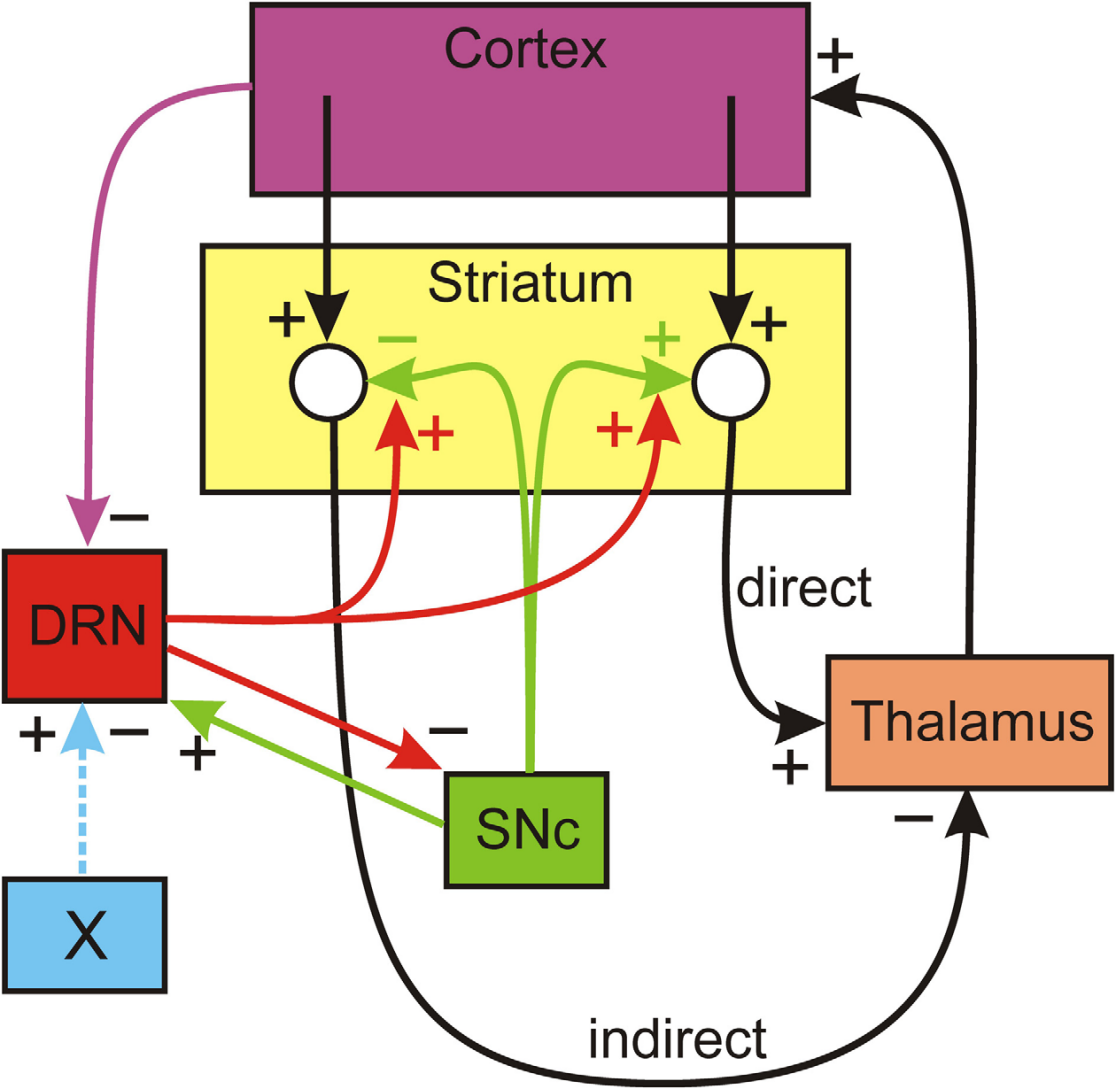
**The influence of the DRN on the direct and indirect pathways.** Dopaminergic neurons of the SNc inhibit the indirect pathway and stimulate the direct pathway from the cortex to the thalamus. Serotonin from the DRN projections to the striatum increase DA release and projections from the cortex inhibit DRN firing. The details of basal ganglia BG circuitry in the direct and indirect pathways is omitted. References for the influences are given in the text. There are many descending projections to the DRN; nucleus X represents one of these that may excite or inhibit the DRN.

The circuit we will consider is shown schematically in Figure 1. Dopaminergic neurons in the SNc innervate the MSNs in the striatum, inhibiting the MSNs in the indirect pathway and stimulating the MSNs in the direct pathway. The DRN sends dense serotonergic projections to the striatum (Vertes, 1991) and it is known that increased concentrations of 5-HT in the striatum facilitate the release of DA from the dopaminergic projections from the SNc (Blandina et al., 1989; Bonhomme et al., 1995; Deurwaerdere et al., 1996). Projections from the thalamus excite cortical neurons. There are numerous projections from higher brain regions to the DRN (Monti, 2010); in particular there are inhibitory projections from medial prefrontal cortex (mPFC) (Celada et al., 2001). In addition, SNc projections excite DRN neurons (DiMatteo et al., 2008) and DRN neurons inhibit SNc neurons (Guiard et al., 2008). Nucleus X stands for some other nucleus that sends excitatory or inhibitory projections to the DRN. This is the basic structure of our model that is indicated schematically in Figure 1; many of the details of the direct and indirect pathways, described in Smith et al. (1998), have been omitted here.

We can now describe verbally how the circuit in Figure 1 allows the serotonergic projection from the DRN to the striatum to maintain the balance between the direct and indirect pathways. Suppose, for some reason, that the firing of the DA neurons in the SNc goes down. This will lower DA in the striatum, which in turn will decrease the firing in the direct pathway and increase the firing in the indirect pathway. Therefore, the thalamus will be more inhibited and the ascending projections to the mPFC will fire less. Thus, the descending projections from the mPFC to the DRN will fire less, thus removing inhibition from the DRN. The increased firing of the DRN neurons will release more 5-HT in the striatum. Finally, the increased 5-HT tone in the striatum increases DA release in response to each action potential, thus partially compensating for the initial decreased DA release.

## Methods

In this section we describe the details of the mathematical model that implements the circuit indicated schematically in Figure 1. The variables of the mathematical model, given in Table 1, are the firing rates (in Hz) corresponding to the nuclei pictured in Figure 1 and the concentrations (in nM) of DA and 5-HT in the striatum. The firing rates can be thought of as average firing rates in the nuclei or as the firing rates of particular neurons in the nuclei that correspond to specific actions.

**Table 1:** **Variables**.

**Name**	**Steady state**	**Explanation**
MI	1.88	Firing rate of striatal spiny neuron in the indirect pathway (Hz)
MD	1.85	Firing rate of striatal spiny neuron in the direct pathway (Hz)
TH	17.5	Firing rate of thalamic neuron (Hz)
CX	26.3	Firing rate of cortical neuron (Hz)
DRN	1.41	Firing rate of dorsal raphe nucleus neuron (Hz)
DA	2.72	Concentration of dopamine in the striatum (nM)
5HT	0.846	Concentration of serotonin in the striatum (nM)
SN	4.47	Firing rate of substantia nigra pars compacta neuron (Hz)

The differential equations satisfied by the variables are given below. They are the simplest equations one can imagine that express the positive and negative influences between the variables shown in Figure 1. There are external input terms. The influences are all simple linear functions of the relevant variables except for the term *G* · (5*HT*) · (*SN*) that expresses the release of DA in the striatum as function of SNc firing rate and of 5-HT concentration in the striatum. Most of the equations have decay terms that guarantee that firing or concentrations go to zero in the absence of input. Of course, the real physiological situation is much more complicated. We have left out important nuclei in the BG and there is no representation of the important dynamics within various nuclei. The purpose of this simple model is merely to allow us to investigate and illustrate the hypothesis about the role of 5-HT in maintaining homeostasis between the direct and indirect pathways.

(1) $$\frac{dMI}{dt}=a_{1c}-a_{1da}\cdot DA-d_{1}\cdot MI$$

(2) $$\frac{dMD}{dt}=a_{2c}+a_{2da}\cdot DA-d_{2}\cdot MD$$

(3) $$\frac{dTH}{dt}=a_{3}+a_{3md}\cdot MD-a_{3mi}\cdot MI-d_{3}\cdot TH$$

(4) $$\frac{dCX}{dt}=a_{4th}\cdot TH-d_{4}\cdot CX$$

(5) $$\frac{dDRN}{dt}=a_{5}-a_{5cx}\cdot CX+a_{5sn}\cdot SN-d_{5}\cdot DRN$$

(6) $$\frac{dDA}{dt}=G\cdot 5HT\cdot SN-d_{6}\cdot DA$$

(7) $$\frac{d5HT}{dt}=a_{7}\cdot DRN-d_{7}\cdot 5HT$$

(8) $$\frac{dSN}{dt}=a_{8}-a_{8drn}\cdot DRN-d_{8}\cdot SN$$

The values of the parameters in the differential equations and their meanings are listed in Table 2.

**Table 2:** **Parameters**.

**Name**	**Value**	**Meaning**
*a*_1*c*_	2.333	Cortical input to medium spiny neurons in indirect pathway
*a*_1*da*_	0.167	Influence of DA on medium spiny neurons in indirect pathway
*d*_1_	1	Decay constant of medium spiny neurons in indirect pathway
*a*_2*c*_	1.167	Cortical input to medium spiny neurons in direct pathway
*a*_2*da*_	0.250	Influence of DA on medium spiny neurons in direct pathway
*d*_2_	1	Decay constant of medium spiny neurons in indirect pathway
*a*_3_	1.667	External drive to thalamus
*a*_3*md*_	3.5	Excitation of direct pathway on the thalamus
*a*_3*mi*_	2	Inhibition of indirect pathway on the thalamus
*d*_3_	0.25	Decay constant of thalamic neurons
*a*_4*th*_	1.5	Influence of thalamus on cortex
*d*_4_	1	Decay constant of cortical neurons
*a*_5_	6.667	External drive to the DRN
*a*_5*sn*_	0.01	Excitatory influence of the SNc on the DRN
*a*_5*cx*_	0.175	Inhibition of the DRN neurons by the cortex
*d*_5_	1.5	Decay constant of DRN neurons
*G*	0.72	The influence per nM 5-HT on DA release in the striatum
*d*_6_	1	Decay constant of DA in the striatum
*a*_7_	1.2	Influence of DRN firing on 5-HT release in the striatum
*d*_7_	2	Decay constant of 5-HT in the striatum
*a*_8_	58.833	External drive of the SNc
*a*_8*drn*_	10	Inhibition of SNc neurons by the DRN
*d*_8_	10	Decay constant of SNc neurons

The values of the parameters were adjusted so that the steady state values of the variables are in ranges that correspond to experimental observations. For example, the steady state concentration of DA in the striatum is 2.72 nM (Segovia and DelArco, 1997; Jones et al., 1998), the steady state concentration of 5-HT in the striatum is 0.846 nM (Knobelman et al., 2001), the DRN firing rate is 1.41 Hz (Feldman et al., 1997), the SNc firing rate is 4.47 Hz (Feldman et al., 1997), the firing rates in the indirect and direct pathways are 1.9 Hz (Mahon et al., 2006), and the thalamic firing rate is 17.5 Hz (Ohara et al., 2007). We have not been specific about which cortical region is projecting to the DRN in our model so we didn't make an effort to put that rate in any particular range.

## Results

In this section we describe some experiments with the model that illustrate and confirm some of the speculations discussed in the Introduction. We will see that the circuit in Figure 1 automatically compensates for changes in the system that would unbalance the direct and indirect pathways. The firing rates discussed can be interpreted as average firing rates of populations of neurons or as firing rates of individual neurons.

### Decrease in SNc firing

Suppose that the average firing rate of SNc cells decreases, for example by cell death as in Parkinson's disease. We can simulate this condition by raising the parameter *d*_8_ from 10 to 17 because this will lower SNc firing at steady state. If we do this, the new steady state values of the variables are given in column A1 of Table 3. The units are Hz except for DA and 5-HT where the units are nM. These new steady state values are to be compared with the baseline steady states given in the column labeled “normal” in Table 3. As one can see, the SNc firing rate falls 49% but DA concentration in the striatum falls from 2.72 to 1.98 a decrease of only 27%. The indirect pathway increases by only 6.6% and the direct pathway decreases by only 10%. This is the homeostatic effect of the DRN projection to the striatum discussed above, and it is easy to understand using Figure 1. As SNc firing decreases, the indirect pathway is more stimulated and the direct pathway is less stimulated. Thus, the thalamus is more inhibited and fires less, so the cortex is less stimulated and fires less. This removes some cortical inhibition from the DRN which fires more, driving the concentration of 5-HT up in the striatum. The enhanced 5-HT concentration in the striatum causes more DA release, which partially compensates for the lower SNc firing rate.

**Table 3:** **Results of simulations no. 1**.

**Name**	**Normal**	**A1**	**A2**	**B**	**C**
MI	1.88	2.00	2.145	2.04	2.04
MD	1.85	1.66	1.448	1.66	1.61
TH	17.5	13.93	9.772	12.84	12.84
CX	26.3	20.90	14.66	19.26	19.26
DRN	1.41	2.02	2.75	2.22	2.20
DA	2.72	1.98	1.124	1.76	1.76
5HT	0.846	1.213	1.648	1.33	0.667
SN	4.47	2.271	1.845	3.66	3.66

To test whether these homeostatic effects really are the result of the circuit in Figure 1, we ran the same simulation as in A1, but now we kept the 5-HT in the gain term [the first term on the right side of equation (6)] constant at its normal value of 0.846 nM. Thus, we have kept the circuit intact but the increase of 5HT in the striatum will not be felt by the DA terminals. The results are shown in column A2. Now, MI increases by 14%, MD decreases by 22% and DA in the striatum decreases by 54%.

### Decrease in the gain *G*

Suppose that the gain, *G*, that governs how much each nM of 5-HT in the striatum increases DA release is cut in half. One could imagine that half the 5-HT receptors on DA terminals in the striatum become ineffective. The new steady state values are are given in column B of Table 3. *MI* increases by only 7.5% and MD decreases by only 13%. The reason is that as the direct and indirect pathways start to become unbalanced the thalamus fires less and thus the cortex fires less. Thus, the corical inhibition is partially withdrawn and the DRN fires a lot more (up 58%) and this causes a 58% rise in striatal 5-HT that partially compensates for the drop in *G*. This is true despite the fact that the SNc firing rate declines because of inhibition from the DRN.

### Decrease in 5-HT release

Suppose that we decrease by 50% the coefficient *a*_7_, which represents the amount of 5-HT released per DRN spike in the striatum. The new steady states are given in column C of Table 3. Although one might expect that 5-HT would decrease by 50% in the striatum, in fact it only decreases by 21%. The reason, as above, is that as the direct and indirect pathways become unbalanced, the thalamus and cortex fire less, so the inhibition of the DRN is partially withdrawn and DRN firing rises 58%, which partially compensates for the drop in the 5-HT release coefficient *a*_7_. As a result, the indirect and direct pathways change by only 7.5 and 15%.

### The effects of SSRIs

The mechanisms by which chronic doses of selective serotonin reuptake inhibitors (SSRIs) relieve depressive symptoms in some patients remain largely unknown. Since many proposed mechanisms involve increases in extracellular 5-HT in projection regions of the raphe nuclei, it was of interest to ask what changes in the circuit in Figure 1 would result from an increase in 5-HT in the striatum. If we decrease the coefficient *d*_7_ in equation (7) then we expect that the steady state value of 5-HT in the striatum would increase, so we decreased *d*_7_ from 2 to 1. The results can be seen in column D of Table 4. The firing rates in the direct and indirect pathways change modestly. But the most dramatic effect is on the firing of the DRN which decreases by 44%. As a result, the concentration of 5-HT only increases from 0.846 to 0.944 nM. It is known that acute doses of SSRIs decrease DRN firing (Gartside et al., 1995; Hajos et al., 1995; El Mansari et al., 2005), so the circuit in Figure 1 gives a another mechanism by which that could occur. Of course, the standard mechanism is that by increasing the extrcellular concentration of 5-HT near the cell bodies in the DRN, the 5-HT1A auto receptors are stimulated and this decreases DRN firing.

**Table 4:** **Results of simulations no. 2**.

**Name**	**Normal**	**D**	**E**	**F1**	**F2**
MI	1.88	1.745	1.749	1.71	2.14
MD	1.85	2.033	2.041	2.1	1.46
TH	17.5	21.1	21.24	22.36	10.02
CX	26.3	31.64	31.86	33.54	15.03
DRN	1.41	0.787	0.795	2.78	0.51
DA	2.72	3.47	3.496	3.73	1.17
5HT	0.846	0.944	0.477	1.67	0.30
SN	4.47	5.096	10.176	3.11	5.38

### Deep brain stimulation of the SNc

Since deep brain stimulation is used to treat a variety of neurological disorders, we asked how stimulation of the SNc would affect the circuit in the model. To do this we lowered the constant *d*_8_ from 10 to 5 which should increase SNc firing. The results can be seen in column E of Table 4; indeed the firing rate of the SNc more than doubles. More interesting is the fact than the firing rate of the DRN decreases by 44% because of the extra excitation of the direct pathway and the extra inhibition of the in direct pathway. It is tempting to wonder whether this is the cause of the the transient acute depression seen in some patients when the SNc is stimulated (Bejjani et al., 1999).

The simulations in A–E show how the network in Figure 1 automatically compensates for “flaws” in the DA and 5-HT systems to try to keep the direct and indirect pathways in balance. Of course, there may be times when it is important to change the balance between the pathways.

### Changing the balance between the direct and indirect pathways

The role of 5-HT in increasing DA release and the circuit in Figure 1 can also be used to unbalance the direct and indirect pathways. Suppose that there is some other nucleus (called nucleus X in Figure 1) that projects to the DRN. The projection may be excitatory or inhibitory, which would correspond to increasing or decreasing the external drive, *a*_5_, to the DRN. If *a*_5_ is *increased* by 50%, the new steady states are as given in column F1 of Table 4. So, the indirect pathway has been decreased by 9% and the direct pathway has been increased by 14%.

Conversely, if we *decrease a*_5_ by 50% the new steady states are as given in column F2 of Table 4. We see that the indirect pathway is increased by 14% and the direct pathway is decreased by 21%. Thus, the brain can control the balance between the direct and indirect pathways via projections to the DRN.

### Phasic cortical input to the direct pathway

**Figure 2:**
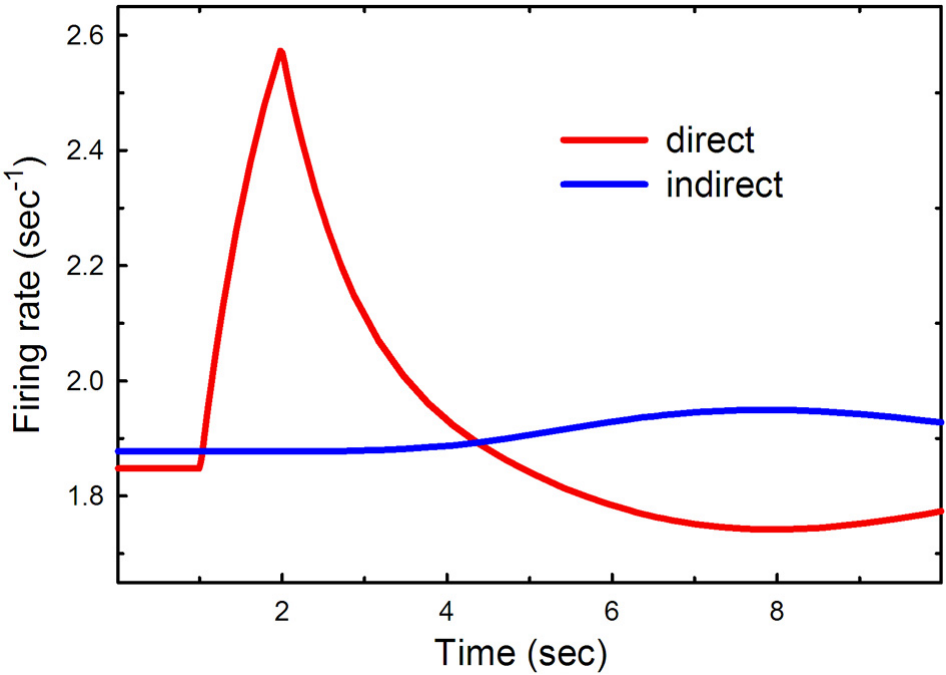
**Phasic cortical input to the direct pathway.** The cortical input to the direct pathway, *a*_2_, was doubled between *t* = 1 s and *t* = 2 s. The red and blue curves show the firing rates as a function of time in the direct and indirect pathways, respectively.

Suppose that there is a burst of cortical input to the direct pathway such as might occur when a particular action is selected and a particular group of striatal neurons in the direct pathway is stimulated. Now we are thinking of Figure 1 as the connection diagram for small groups of neurons in each nucleus that correspond to this action. To test the result in the model we double the cortical input, *a*_2_, to the direct pathway for one second between *t* = 1 s and *t* = 2 s. The result can be seen in Figure 2.

There is a substantial increase in firing in the direct pathway that decays in about 4 s. Notice that this is followed by several seconds in which the direct pathway is inhibited and the indirect pathway is increased. This is a result of the feedback pathway via the DRN depicted in Figure 1. It is tempting to speculate that this inhibition may play a functional role in action-selection by turning off the direct pathway after it has been stimulated.

## Discussion

The purpose of the simple model in this paper is to illustrate and investigate various homeostatic mechanisms arising from the DRN projections to the BG. It is clear that the model is highly simplified and that many important physiological details have not been considered. This paper is based on the fact that direct pathway MSNs express D1 receptors and are stimulated by DA, while MSNs in the indirect pathway express D2 receptors and are inhibited by DA. There are other sub-circuits of the BG, such as the hyperdirect loop (Leblois et al., 2006), and there are other important serotonergic projections to the BG such as the moderately dense projection from the DRN to the globus pallidus. There are also other interactions between 5-HT and DA in the BG that we have not discussed here. Boureau and Dayan (2011) discuss the opponency between DA and 5-HT in behavior. Dopamine is important for changes in synaptic strength in the cortical striatal pathway (Gerfen and Surmeier, 2011). Further, there are differences between the direct and indirect pathways other than the different dopamine receptor types. For example, indirect pathway MSNs are more excitable, an effect thought to be due to differences in morphology (Gerfen and Surmeier, 2011). It is known in animal models that there is rapid hyper-innervation of the striatum by 5-HT terminals after SNc lesions Maeda et al. (2003). This would increase the gain, *G*, in our model and also provide homeostasis of extracellular DA.

It is not surprising that the BG, a regulatory biochemical and electrophysiological network of enormous importance for many aspects of human behavior, would have many built-in homeostatic mechanisms. Such mechanisms buffer the functions of the BG in the face of normal biological variations in the parts and also help to keep the BG functional in the face of large environmentally caused perturbations such as drug use and cell death. One of the most important such homeostatic mechanisms is the “passive stabilization” of DA in the striatum after cell death in the SNc. It is well known that the tissue content of DA in the striatum declines more or less proportionally to cell death in the SNc (Bezard et al., 2001; Dentresangle et al., 2001; Bergstrom and Garris, 2003), but that the extracellular level of DA in the striatum remains almost constant until 80 or 90% of the cells of the SNc have died. Many active compensatory mechanisms were proposed, see for example Hornykiewicz (1966), to explain this remarkable homeostasis. However, a very simple explanation was proposed in Bergstrom and Garris (2003). As SNc cells die, less DA is released into the striatum but there are also proportionally fewer dopamine reuptake transporters, so the concentration in the extracellular space should remain constant. This proposal by Bergstrom and Garris was verified by the current authors using mathematical modeling in Reed et al. (2009), where it is also explained why the homeostasis breaks down when more than 80% of the SNc cells have died.

In this paper we show how the circuitry in Figure 1 automatically leads to several other homeostatic mechanisms. In Results A we showed that in the face of decreased SNc cell firing the level of 5-HT in the striatum will go up and increase the release of DA per action potential, partially compensating for the loss of SNc cell firing. Thus the circuit tends to keep the balance between the direct and indirect pathways. We note that this is consistent with the finding that the DRN fires more in animal models of PD (Zhang et al., 2007; Kaya et al., 2008; Wang et al., 2009). In Results B we showed that if the gain that measures the influence of 5-HT on DA release decreases (for example by receptor loss), then the circuit automatically partially compensates by increasing the firing rate of DRN neurons and thus releasing more 5-HT. In Results C we showed that if there is a drop in 5-HT release in the striatum, the circuit will automatically compensate by increasing DRN firing. All of these effects depend, of course, on the descending inhibitory projections from the cortex to the DRN, which in our model we assume are coming from the mPFC (Celada et al., 2001). So, these results give a reason for the descending inhibitory projections from the cortex to the DRN.

We also show (Results D) that increasing 5-HT in the striatum, as caused by an SSRI for example, would depress firing in the DRN. And, similarly, deep brain stimulation of the SNc would also depress DRN firing (Results E) and may explain the transient depression seen in some DBS patients.

There are times when it might be important to overcome one of these homeostatic mechanisms, for example by changing the balance between the direct and indirect pathways for a subset of neurons involved in choosing a particular action. We showed in Results F how this can be done by changing the input to the DRN, in our example by excitatory or inhibitory input from a hypothesized nucleus X. It is tempting to think that this gives a possible explanation of the plethora of descending projections to the DRN (Monti, 2010).

Our intention was to use our simple model to discuss possible roles of 5-HT in the striatum. Though our results are suggestive, the model depends on the circuitry that we hypothesize in Figure 1. And, therefore, firm conclusions depend on experimental confirmation.

### Conflict of interest statement

The authors declare that the research was conducted in the absence of any commercial or financial relationships that could be construed as a potential conflict of interest.
